# "Present and future of immunotherapy in Neuroendocrine Tumors"

**DOI:** 10.1007/s11154-021-09647-z

**Published:** 2021-04-14

**Authors:** Manuela Albertelli, Andrea Dotto, Federica Nista, Alessandro Veresani, Luca Patti, Stefano Gay, Stefania Sciallero, Mara Boschetti, Diego Ferone

**Affiliations:** 1 Endocrinology Unit, IRCCS AOU San Martino, Genoa, Italy; 2 Endocrinology Unit, Department of Internal Medicine and Medical Specialties (DiMI), Centre of Excellence for Biomedical Research (CEBR), University of Genoa, Genoa, Italy; 3 Medical Oncology Unit 1, IRCCS AOU San Martino, Genoa, Italy

**Keywords:** Immunotherapy, Immune checkpoint inhibitors, Neuroendocrine tumors, Neuroendocrine neoplasia, Merkel Cell Carcinoma, Small Cell Lung Cancer

## Abstract

Immunotherapy, so promising in many neoplasms, still does not have a precise role in the treatment of neuroendocrine neoplasms (NENs). In this article, we provide an overview on the current knowledge about immunotherapy with immune checkpoint inhibitors (ICIs) applied to NENs, evaluating future perspectives in this setting of tumors.

Evidence so far available for ICIs in gastroenteropancreatic (GEP)-NENs is definitively not as robust as for other tumors such as Small Cell Lung Cancer or Merkel Cell Carcinoma. In fact, with regard to the well-differentiated forms of NENs (NETs), the results obtained nowadays have been disappointing. However, the near future, might reserve interesting results for ICIs in GEP-NEN from a total of nine different ICI drugs, used throughout 19 randomised controlled trials. Such numbers highlight the growing attention gathering around NENs and ICIs, in response to the need of stronger evidences supporting such therapy.

For the future, the most important aspect will be to study strategies that can make NETs more susceptible to response to ICI and, thus, enhance the effectiveness of these treatments. Therefore, the combination of conventional therapy, target therapy and immunotherapy deserve attention and warrant to be explored. A sequential chemotherapy, possibly inducing an increase in tumor mutational burden and tested before immunotherapy, could be a hypothesis deserving more consideration. A radiation treatment that increases tumor-infiltrating lymphocytes, could be another approach to explore before ICIs in NENs. Equally essential will be the identification of biomarkers useful for selecting patients potentially responsive to this type of treatment.

## Introduction

While in the past the field of cancer therapy was dominate by surgery and radiotherapy associated with chemotherapy [1, 2], in the last decades research has provided new treatment strategies, such as target therapies. Target therapy, understood as use of drugs or other substances that targets specific molecules to arrest the growth and spread of cancer cells [3], has sharply revolutionized the outcomes of different types of cancer.

Currently, another intriguing weapon available in cancer therapy is represented by immunotherapy, based on the stimulation of the immune system against cancer cells through the introduction of cytokines and antibodies (passive immunotherapy) or the introduction of vaccines and immune cells themselves (active immunotherapy) [4]. Immune evasion mechanisms have a pivotal role for tumor cell proliferation and growth [5, 6]. The Cytotoxic T-Lymphocyte Antigen 4 (CTLA-4) [7] and the programmed death-1 (PD-1) and programmed death-1 ligand (PD-L1) [8] represent a key point regarding escape from immune surveillance by cancer cells, and this reason has led to the development of antibodies against these molecules. These antibodies, named ‘immune checkpoint inhibitors’ (ICIs), have opened a new era in oncology [9, 10]. ICIs proved one of the most effective therapeutic approaches for several cancers. However, many patients are unresponsive to ICIs [11] and these immunotherapies can produce serious non-specific systemic inflammation and autoimmune side effects [12]. Therefore, the study of the tumor microenvironment has become fundamental [13] with the aim of identifying biomarkers to select the patients who can benefit most from these treatments.

Immunotherapy, so promising in many neoplasms, still does not have a precise role in the treatment of neuroendocrine neoplasms (NENs).

NENs constitute a heterogeneous group of rare tumors, which arise from enterochromaffin cells and can present throughout the body, however, in most cases, they are localized in the gastrointestinal tract, pancreas and thorax [14]. A rate amounting around 11–22% of all NENs are defined as unknown primary origin when their primary tissue of origin has not been identified with standard diagnostic work-up. Delineating the primary site of origin has important implications for selecting the appropriate treatment and overall prognosis. The small bowel, followed by the lung and pancreas are the most prevalent primary sites of origin in case of NEN with unknown primary. [15]

NENs display a highly variable biological features, clinical course and prognosis, making prediction of survival difficult. The most recent WHO 2019 classification [16]*,* based on the WHO 2010 classification and extending the WHO 2017 classification [17], has established the importance of classifying gastroeneteropancreatic (GEP) NENs considering the primary site localization, the morphological differentiation, and the grading. According with these affirmations, GEP-NENs are graded into three different categories taking into account the Ki67 proliferation index. The real novelty of the last WHO 2019 classification is the G3 category, characterized by a Ki67 > 20% and a well differentiation morphology, extended to all GEP-NENs. Furthermore, it worth considering neuroendocrine carcinomas (NECs) represented by a Ki67 proliferation index > 20% and poorly differentiated.

The cornerstone of treatment in well-differentiated neuroendocrine tumors (NETs) is based on surgery, local ablative treatments, antisecretory and antiproliferative drugs, such as somatostatin receptor ligands (SRLs), peptide receptor radionuclide therapy (PRRT), and target therapies, while high-grade NECs benefit mostly of platinum-based cytotoxic chemotherapy [18]. However, new therapeutic strategies are being studied for the treatment of NENs, such as immunotherapy. In particular promising results have been especially observed in the treatment of two of most aggressive NENs with ICIs [19]: Merkel cell carcinoma (MCC), a rare very aggressive NET of the skin, and small cell lung cancer (SCLC). Few other types of neuroendocrine neoplasms are beginning to show interesting results [20].

In this article, we provide an overview on the current knowledge about immunotherapy applied to NENs, evaluating future perspectives in this setting.

## Methods

We performed a literature search by MEDLINE (PubMed database) and we also considered the trials registered on *clinicaltrials.gov* to identify potentially relevant articles on immunotherapy with ICIs and NENs of any grade and primary site. The search was last updated 31 January 2021. The search strategy included the following terms “immunotherapy” OR “immune checkpoint inhibitor” OR “immune checkpoint blockade” OR “spartalizumab” OR “pembrolizumab” OR “toripalimab” OR “nivolumab” OR “ipilimumab” OR “atezolizumab” AND:

- GEP-NENs section: “gastroeneteropancreatic neuroendrocine neoplasms” OR “GEP-NENs” OR “gastroenteropancreatic neuroendocrine tumors”
- thoracic NENs section: “typical carcinoid” OR “atypical carcinoid” OR “large cell neuroendocrine carcinoma (LCNEC)” OR “SCLC”
- MCC section: “Merkel Cell Carcinoma” OR “ MCC”

Only articles published in English were considered. Additional studies were identified by reviewing the references of all selected articles. The methods of potentially eligible studies were assessed independently by three reviewers (MA, AD, FN).

## Gastroeneteropancreatic neuroendocrine neoplasms (GEP-NENs)

The incidence of NENs has risen in the last few decades*.* Especially, as for prevalence, GEP-NETs are the second neoplasm of the gastro-intestinal tract [21]. GEP-NENs arise in secretory cells of the gastrointestinal tract (GI-NENs) and pancreas (panNENs) [22, 23]. As for treatment, in well-differentiated GEP-NETs the first choice is local surgical treatment, when applicable. In case of metastatic NETs or surgical approach unenforceable, antiproliferative and antisecretory therapy with SRLs are recommended [18, 24]. Moreover, other strategies such as PRRT has been demonstrated effective in prolonging progression-free survival (PFS) especially in patients with GI-NETs [25]. Everolimus, sunitinib, and chemotherapy are further treatments investigated and approved for GEP-NENs. As regards GEP-NECs, cytotoxic chemotherapy platinum-based with etoposide is the most common available approach [26, 27]. It’s worth considering the poor prognosis of these carcinomas with a median of about 7.5 months [28]*.* Furthermore, the therapeutic landscape for NENs has been evolving constantly and several clinical trials are ongoing to characterize immunotherapy as an emerging therapeutic strategy. In particular, ICIs, using anti–PD-1, anti–PD-L1 and anti-CTLA-4 agents, has been investigated in GEP-NENs in the last few years. Several studies have evaluated the expression of these checkpoints molecules in NETs of different grades and in NECs [29, 30]. The potential a given NEN to respond to ICI is still largely unknown. Immunohistochemical (IHC) evaluation of PD-L1 expression in the tumor microenvironment and its role in predicting response to ICIs is a very burning topic. Moreover, a high tumor mutational burden (TMB) has been found associated to an increased benefit of ICIs [31–33] and was advocated as a possible useful biomarker to select potential responders to these drugs.

The importance of the immune microenvironment in patients with NETs has been established in the last years [34]. Immune cell infiltration is only documented in GEP-NETs, and overall, it appears to be higher in panNETs rather than in midgut NETs, perhaps as a result of the higher TMB of panNETs [35, 36]. Morever, in another series of NENs, potentially immunosuppressive regulatory T cells (Treg) were present in 55% of intermediate/high-grade tumours, whereas only 16% of low-grade metastatic NENs showed intratumoral Treg (P = 0.02). [37]. Nevertheless, more knowledge about the composite immune landscape of these heterogeneous tumors needs to be gained in order to clarify the prognostic implication that have these NENs features. [38].

Furthermore, even prognostic evidences linked with the expression of immune checkpoint molecules PD-L1 and PD-1 are still unclear and debated across different studies [39–42]. There are some data in the literature showing that PD-L1 expression was significantly correlated to a higher WHO grade of NENs [39, 42] and also with a poor PFS and overall survival (OS). On the contrary, others authors have found that PD-L1 expression did not correlate with grade or prognosis [40, 43]. In fact, recently, the expression of PD-L1 was investigated in a large cohort of patients with G3 GEP-NEN and only 10% of these tumors expressed PD-L1 and those lesions, with positive PD-L1 immunoreactivity in tumoral cells, were all poorly differentiated cases [43]. In this study no correlation was found between PD-L1 immunoreactivity and clinical parameters evaluated, such as age, sex, primary site, PFS and OS. Anyway, the design of the study did not include treatment intervention with ICIs, therefore the clinical outcome and the response to therapy could not be correlated with the expression of PD-L1 in this series [43]*.*

As reported in different studies, TMB is typically low in well-differentiated lesions (NET G1 or G2) and higher in poorly differentiated carcinomas (G3), [34, 44].

Globally, all these studies have aroused new perspectives to initiate clinical trials aimed to identify the efficacy of ICIs in NENs.

### Pembrolizumab

The non-randomized, phase 1b trial KEYNOTE-028 investigated the safety and efficacy of the PD-1 inhibitor pembrolizumab, as monotherapy, in a large cohort of patients with advanced solid tumors with positive PD-L1 expression [45] As for NENs, 16 patients with pancreatic NEC were considered, one patient had objective response and 14 of them experienced stable disease. Interestingly, encouraging data emerged from the six-months follow-up demonstrating a PFS rate of 40%. Furthermore, at the 12-months evaluation, the PFS rate was of 27% and OS rate of 87%. The following phase 2 KEYNOTE-158 trial, considering GEP-NETs, partial response was present in 3 patients with panNET and in 1 subject with rectal NET after a median follow-up of 24 months [46].

### Spartalizumab

Spartalizumab is a humanized anti-PD1 monoclonal antibody, evaluated in a phase II, multicenter, single-arm study enrolling patients with well-differentiated metastatic G1/G2 NET (32 GI-NET; 33 panNET) and GEP-NEC. In the 21 patients with GEP-NECs a higher expression of PD-L1 in immune cells was observed respect than those with GEP-NETs. As for the NETs cohort, the objective response rate (ORR) was 3,1% in GI-NET and 3% in panNET. These data did not accomplish the success label fixed as ORR ≥ 10% [47]*.* In the GEP-NEC group, ORR was 4.8% (95% CI: 0.1, 23.8) and the 12-month overall survival was 19.1%. Interestingly, the ORR was higher in patients with higher PD-L1 expression or more CD8 + cells infiltration at baseline evaluation. Furthermore, spartalizumab determined limited adverse effects (observed in less than 50% of patients). In the GEP-NEC cohort less than 20% of subjects presented increased liver enzymes.

### Toripalimab

Toripalimab is a humanized IgG4 antibody with human PD-1 receptor as target. This molecule was approved few years ago as second-line treatment in metastatic melanoma. A phase 1b trial investigated its efficacy in patients with NENs recurring or metastatic after first-line therapy. Toripalimab schedule therapy was 3 mg/kg once every two weeks. [48]. In the cohort of 40 subjects, ORR was 20% and the median durability of response (DOR) was 15.2 months. Interestingly, in tumors characterized by PD-L1 expression ≥ 10% ORR was 50% while in those with PD-L1 < 10% ORR was 10.7% (p = 0.019).

### Ipilimumab and nivolumab

The prospective, open-label, multicenter phase II clinical trial DART (Dual Anti-CTLA-4 and Anti-PD-1 Blockade in Rare Tumors, the trial is still ongoing, NCT02834013) investigated the efficacy and safety of the combined immunotherapy: ipilimumab plus nivolumab across multiple rare tumor cohorts. [49]*.* Ipilimumab combined with nivolumab were tested in a cohort of 32 patients with any grade of non panNETs. As for GI-NETs, 15 patients were enrolled and 18 out 32 patients had high-grade tumors. Results demonstrated a 44% ORR in patients with NEC versus 0% in low and intermediate grade neoplasms. This combined therapy was investigated in another phase 2 trial, involving 29 patients with advanced NETs: overall, the ORR was 24% and as for panNETs 43% of patients experienced an objective response [50].

## Future perspective in GEP-NENs

Evidence so far available for ICIs in GEP-NETs is definitively not as robust as for other tumors such as SCLC or MCC. We, therefore, had to take a further look to what the near future might reserve for ICIs in GEP-NET and NEC through an in-depth research on the *clinicaltrials.gov* database, considering only phase II or III studies, evaluating every Food and Drug Administration (FDA) approved ICIs molecule and not approved ones as well. Our research yielded 42 results, of which 19 were deemed relevant for our purpose; the results are summarized in Table 1; the considered drugs are used either in monotherapy, or in association with another ICIs, with Tirosin Kinase Inhibitors (TKI)/other target therapy, or with SRLs, or PRRT, or chemotherapy.

**Table 1:** Ongoing RCT for ICIs in GEP-NENs

ClinicalTrials.gov Identifier	Molecule	Study phase	Assigned intervention	Primary outcome(s)	Estimated enrollment, n	Estimated study completion date	Trial status
NCT03043664* Phase Ib/II Study of Pembrolizumab With Lanreotide Depot for Gastroenteropancreatic Neuroendocrine Tumors	Pembrolizumab	Phase Ib—II	Pembrolizumab 200 mg intravenous every 3 weeks and lanreotide depot 90 mg subcutaneous every 3 weeks	Objective response rate	22	June 1, 2021	Active, not recruiting
NCT03901378 A Phase II Trial of Pembrolizumab in Combination With Cisplatin or Carboplatin and Etoposide in Chemotherapy naïve Patients With Metastatic or Unresectable High Grade Gastroenteropancreatic or Lung (Excluding Small Cell) Neuroendocrine Carcinoma	Pembrolizumab	Phase II	Pembrolizumab 200 mg with Carboplatin or Cisplatin plus etoposide. Repeat 4–6 cycles followed by Pembrolizumab alone until progression or intolerance for up to 2 years	Progression-free survival	-	-	Withdrawn (Lack of accrual)
NCT03136055** A Pilot Study of Pembrolizumab-based Therapy in Previously Treated High Grade Neuroendocrine Carcinomas	Pembrolizumab	Phase II	Part A: pembrolizumab 200 mg every three weeks Part B: pembrolizumab 200 mg every three weeks and, either - irinotecan 125 mg/m2 in a two weeks on, one week off format in 3 week cycles, or - paclitaxel 80 mg/m2 every week	Overall response rate	36	July 1, 2023	Active, not recruiting
NCT03278405 A Pilot Study of Avelumab in Unresectable/Metastatic, Progressive, Poorly Differentiated Grade 3 Neuroendocrine Carcinomas	Avelumab	Phase I—II	Avelumab 10 mg/kg once every 2 weeks, until occurence of progressive disease, unacceptable toxicity, or any of the other criteria for withdrawal listed in the protocol	Overall response rate	10	March 12, 2020	Completed
NCT03147404 Phase II Study of Avelumab in Metastatic Gastronetro-pancreatic (GEP) Neuroendocrine Carcinoma (NEC, WHO Grade 3)as Second-line Treatment After Failing to Etoposide + Cisplatin: Integration of Genomic Analysis to Identify Predictive Molecular Subtypes (MS100070-0177)	Avelumab	Phase II	Avelumab 10 mg/kg every 2 weeks	Best response	14	July 22, 2019	Completed
NCT03591731 A GCO Trial Exploring the Efficacy and Safety of Nivolumab Monotherapy or Nivolumab Plus Ipilimumab in Pre-treated Patients With Advanced, Refractory Pulmonary or Gastroenteropancreatic Poorly Differentiated Neuroendocrine	Nivolumab / Nivolumab + Ipilimumab	Phase II	[arm A]: Nivolumab 3 mg/kg every 2 weeks [arm B]: Nivolumab 3 mg/kg every 2 weeks + Ipilimumab 1 mg/kg every 6 weeks	Objective response rate	180	September 2023	Recruiting
NCT04525638 A Phase II Single Arm Trial Evaluating the Preliminary Efficacy of the Combination of 177Lu-DOTATATE and Nivolumab in Grade 3 Well-differentiated Neuroendocrine Tumours (NET) or Poorly Differentiated Neuroendocrine Carcinomas (NEC)	Nivolumab	Phase II	Nivolumab 240 mg and 7.4 GBq 177Lu-DOTATATE intravenously as a 4-h infusion	Overall response rate	30	September 30, 2024	Recruiting
NCT03352934*** A Phase II, Open-label, Multicenter Trial to Investigate the Clinical Activity and Safety of Avelumab in Patients With Advanced, Metastatic High Grade Neuroendocrine Carcinomas NEC G3 (WHO 2010) Progressive After Chemotherapy	Avelumab	Phase II	10 mg/kg Avelumab every 2 weeks until documented disease progression (PD), unacceptable toxicity, or any criterion for treatment withdrawal are met	Disease control rate	60	January 2024	Active, not recruiting
NCT04079712 A Phase 2 Study of XL184 (Cabozantinib) in Combination With Nivolumab and Ipilimumab for the Treatment of Poorly Differentiated Neuroendocrine Carcinomas	Nivolumab + ipilimumab	Phase II	Cabozantinib on days 1–21 of cycles 1–4 and days 1–28 of subsequent cycles; Nivolumab on day 1, and ipilimumab on day 1 of cycles 1–4 only Treatment repeats every 21 for 4 cycles then every 28 days for subsequent cycles in the absence of disease progression or unacceptable toxicity	Overall response rate	30	October 1, 2021	Recruiting
NCT03190213 Pembrolizumab for the Treatment of Recurrent High Grade Neuroendocrine Carcinoma	Pembrolizumab	Phase II	Pembrolizumab 200 mg every 3 weeks until disease recurrence or discontinuation due to unacceptable toxicity for a maximum of 2 years	Overall response rate	6	March 11, 2019	Terminated (discontinued)
NCT03980925 A Phase II Study of Platinum-doublet Chemotherapy in Combination With Nivolumab as First-line Treatment in Subjects With Unresectable, Locally Advanced or Metastatic G3 Neuroendocrine Neoplasms (NENs) of the Gastroenteropancreatic (GEP) Tract or of Unknown (UK) Origin	Nivolumab	Phase II	[Induction Phase]: Nivolumab 360 mg + Carboplatin + Etoposide on days 1-3D, all every 3 weeks up to 6 cycles followed by Nivolumab 480 mg for 24 months or until PD, death or toxicity [Maintenance Phase]: Nivolumab 480 mg every 4 weeks for 2 years	Overall survival rate	38	December 2022	Recruiting
NCT03290079 Phase II Study of Pembrolizumab and Lenvatinib in Advanced Well-differentiated Neuroendocrine Tumors	Pembrolizumab	Phase II	Pembrolizumab 200 mg every 3 weeks	Objective response rate	30	December 2023	Suspended
NCT03728361**** A Phase II, Multi-Cohort Trial of Combination Nivolumab and Temozolomide in Recurrent/Refractory Small-Cell Lung Cancer and Advanced Neuroendocrine Tumors	Nivolumab	Phase II	Nivolumab on day 1 of a 28 day cycle + temozolomide on days 1–5. Courses repeat every 28 days in the absence of disease progression or unacceptable toxicity	Objective response rate	53	December 31, 2021	Recruiting
NCT03074513***** A Phase II, Single-Arm Open-Label Study of the Combination of Atezolizumab and Bevacizumab in Rare Solid Tumors	Atezolizumab	Phase II	Atezolizumab + Bevacizumab on day 1. Courses repeat every 21 days in the absence of disease progression or unacceptable toxicity	Objective response	164	March 31, 2021	Active, not recruiting
NCT03475953 A Phase I/II Study of Regorafenib Plus Avelumab in Solid Tumors	Avelumab	Phase I—II	Phase 1 and Phase 2: Avelumab every 2 weeks starting at Cycle 1 Day 15. Regorafenib (oral) once daily for three weeks on/one week off	Phase I: Recommended dose of regorafenib Phase II: objective response; progression-free rate	482 (though only one cohort out of ten is made by GEP-NETs)	May 2022	Recruiting
NCT03095274****** A Phase II Study of Durvalumab (MEDI4736) Plus Tremelimumab for the Treatment of Patients With Advanced Neuroendocrine Neoplasms of Gastroenteropancreatic or Lung Origin (the DUNE Trial)	Durvalumab + Tremelimumab	Phase II	Durvalumab 1500 mg for 12 months in patients ≥ 30 kg. durvalumab 20 mg/kg for patients < 30 kg Tremelimumab 75 mg for up to 4 doses/cycles in patients ≥ 30 kg. remelimumab 1 mg/kg for patients < 30 kg	Clinical Benefit Rate	126	July 2021	Recruiting
NCT04579757 An Open-Label Phase Ib/II Study of Surufatinib in Combination With Tislelizumab in Subjects With Advanced Solid Tumors	Tislelizumab	Phase Ib—II	Part 1 (dose escalation): surufatinib administered orally once daily + tislelizumab 200 mg intravenous infusion every 3 weeks Part 2 surufatinib at the Recommended Phase 2 Dose selected in Part 1 + tislelizumab 200 mg every 3 weeks	Dose limiting toxicity; Objective response rate	120	April 30, 2023	Recruiting
NCT03365791******* Modular Phase 2 Study to Link Combination Immune-therapy to Patients With Advanced Solid and Hematologic Malignancies. Module 9: PDR001 Plus LAG525 for Patients With Advanced Solid and Hematologic Malignancies	Spartalizumab	Phase II	PDR001 (spartalizumab) and LAG525 will be administered via i.v. infusion over 30 min once every 3 weeks. LAG525 will be given first followed by PDR001	Clinical Benefit Rate	76	September 17, 2020	Completed

*Initial results posted [48]

**Initial results posted [53]

***Initial results posted [49]

****Initial results posted [50]

*****Initial results posted [52]

******Initial results posted [54]

*******Initial results posted [51]

A total of nine different ICIs drugs are used throughout the 19 Randomised Controlled Trials (RCTs). Such numbers highlight the growing attention gathering around NENs and ICIs, in response to the need of stronger evidences supporting such therapy.

Some of the reported trials have, however, already produced initial results, most of them in the form of abstracts/interim analyses: we retrieved seven initial reports from seven different RCTs, the majority of which showing promising results.

Four analyses report a clinical benefit rate (CBR) > 30% [51–54]. The first [51] is derived from *NCT03043664*, which investigates pembrolizumab plus SRLs in 22 patients with low/intermediate grade GEP-NETs. With this schedule around 40% of patients achieved stable disease (SD). Relevant antitumor activity was also described for avelumab, tested in 29 patients [52] deriving from *NCT03352934*, with either NEC or NET (mostly GEP), that obtained a disease control rate (DCR) of 32%. Nivolumab combined with temozolomide showed promising results in an interim analysis [53] from *NCT03728361*: out of 12 patients (7 GEP-NETs and 5 pulmonary carcinoids) 25% showed partial response (PR), 67% SD and 8% progressive disease (PD), although the median follow-up was short (approximately 7 months) and the cohort very limited. Lastly, according to an interim analysis [54] from *NCT03365791*, spartalizumab, administered with LAG525 (an antibody to LAG-3), achieved an astounding 86% of clinical benefit rate at 24 weeks in a GEP-NET cohort. With the exception of nivolumab plus temozolomide, the majority of the above mentioned analyses reported a good to an excellent tolerability as well.

More modest results are reported in another interim analysis [55] of *NCT03074513*, which aimed at defining the activity of atezolizumab plus bevacizumab in panNET and extra panNETs, achieving a confirmed objective response rate of 20% and 15% (95% CI 3–38%), respectively. This last association was also well tolerated. Worse results were, instead, reported in 14 patients with progressive NECs treated with pembrolizumab alone (partial results from *NCT03136055*). Pembrolizumab was deemed ineffective in this small cohort [56]. Similarly, durvalumab in combination with tremelimumab was almost ineffective the abstract [57] derived from *NCT03095274:* a global cohort of 123 patients was divided in subgroups considering the primary origin and grade (GI NETs, panNETs, GEP NECs and lung carcinoids, 123 patients in total) where a CBR > 30% at median follow-up of 10.8 months was observed only in the panNET group. Nor safety or tolerability concerns were registered.

As already mentioned, the results achieved in these RCTs should be considered partial because derived from studies including often a too low number of patients. However, considering the overall promising preliminary results observed, final results are eagerly waited for.

## Lung neuroendocrine neoplasms

Lung NENs are a group of rare and heterogenous pulmonary tumors classified into four different histological groups following the 2015 WHO lung NEN classification [58]. Well-differentiated lung NENs are typical (TC) and atypical (AC) carcinoids often characterized by indolent clinical behavior. On the other hand, pulmonary high-grade NENs are SCLC and large-cell neuroendocrine carcinoma (LCNEC). Briefly, TC is the most frequent lesion among the four histological groups and have a metastatic potential in up to 15% of patients with a median time to recurrence of 4 years. AC presents metastatic spread in up to one half of cases and has a median time to recurrence of 1.8 years. As for advanced lung NET, median survival from diagnosis is about 6–7 years [59]. Concerning treatment, it worth considering a different approach based on the histological group and patients tailored. Since now, few trials have been designed for lung NETs specifically, therefore the therapeutic management largely derives from studies in patients with GEP-NENs [60]. In this view, the emerging immunotherapy strategies might represent a new base to change the therapeutic scenario of this group of NENs. Particularly, immunotherapy has become a paradigm shift in the treatment of SCLC. The presence of lymphocyte infiltration in lungs NENs have not been fully investigated so far. Researchers have obtained discrepant results as for the association between the host immune response to lymphocyte infiltration and the prognosis of lung NENs, especially in terms of OS and PFS [29].

### Lung carcinoid tumors: typical and a typical (TC and AC)

TC and intermediate-grade AC are heterogenous NENs whose treatment mostly derived from studies designed for GEP-NENs and SCLC. Nowadays, surgery is considered the first-choice treatment in local disease, whereas there is a lack of internationally approved therapies in case of metastatic carcinoids. Recently, immunotherapy has become a promising therapeutic option based on the emerging results regarding PD-1 and PD-L1 expression in lung NETs. It is worth quoting a recent paper enrolling 131 patients with TC and 37 with AC, in which immunohistochemistry was performed to detect the expression of PD-L1, PD-1, and CD8. As a result, PD-1 expression was present in 7% of TC samples and no AC expressed PD-L1. Overall, the study pinpointed that carcinoids showed a low expression of both PD-1 and PD-L1. However, PD-L1 presence was strickling associated with mediastinal lymph-node metastasis at baseline. In this view, immunotherapy might represent a possible therapeutic option in the management of these neoplasms, undoubtedly to be investigated with further studies [61]. In this scenario, a recent case report described a patient with metastatic AC and persistent mediastinal lymphadenopathy refractory to first line treatment with platinum-based chemotherapy and etoposide. According with the results from the CheckMate-032 trial in SCLC, the combined therapy with nivolumab and ipilimumab was undertaken with a success tumor response [62].

Furthermore, the nonrandomized phase 1b KEYNOTE-028 trial, evaluating a cohort of 25 patients with PD-L1–positive TC or AC treated with pembrolizumab, showed ORR of 12%. However, a complete response (CR) was not observed in any patients of this cohort, whereas 15 subjects had stable disease, and 7 presented a progressive disease. At the 12 months follow-up, PFS rate was 27% and ORR 65%. Median OS was 21.1 months (9.1 to 22.4 months) [45].

Moreover, spartalizumab was evaluated in a phase II trial enrolling 116 patients with advanced NETs, of different primary origin, and GEP-NEC, in progression after previous treatments. As regards well-differentiated NETs, the median follow-up was 7.6 months, with an overall ORR of 7.4%. Interestingly, thoracic NETs had a higher ORR (20%). In the thoracic carcinoid cohort, stable disease was reached in 53.3%. PD-L1 expression was higher in GEP-NECs compared to the thoracic carcinoids (43% vs. 19%) [47].

The ongoing trial dual anti-CTLA-4 and anti-PD-1 blockade in rare tumors (DART SWOG 1609), has gathered 32 patients with non-pancreatic NENs, and 6 of them with lung as primary origin. These patients were treated with nivolumab combined with ipilimumab and the overall ORR observed was 25%. Whereas, considering low-intermediate grade neoplasms, ORR was 0% [49].

### Large cell neuroendocrine carcinoma (LCNEC)

LCNEC is a rare carcinoma and accounts for less than 1% of all lung tumors. It has a poor prognosis, with 5-year overall survival rates of about 13–57%. Treatment strategies include surgical approach, radiotherapy, and cytotoxic chemotherapy. Evidences of immunotherapy in these neoplasms are scares and derive especially from cases reports and retrospective analysis. Briefly, an interesting case report pinpointed the remarkable tumor response to nivolumab in a second-line setting, in a young woman with a locally advanced LCNEC (cT4 cN2 cM0 at baseline vs. ycT2a ycN0 ycM0 after 14 cycles) [63]. Similarly to this case, in the literature are present other papers highlighting the response to PD-1 inhibitors, especially nivolumab, as second- or third-line treatment in patients with LCNEC. The larger analysis available, included 10 patients with advanced LCNEC (8 patients had a stage IV disease) treated with nivolumab or pembrolizumab as second-line or further (first line therapy was platinum-based chemotherapy). Overall, 60% of patients had a partial response and in one case a stable disease was reached. Median PFS was 57 weeks and 80% of patients stopped immunotherapy because of disease progression and only one for a pulmonary interstitial pneumonia [64, 65]. There are not significant evidences regarding immunotherapy with PD-1 inhibitors and/or CTLA4 inhibitors as first-line approach in LCNEC.

### Small cell lung cancer (SCLC)

SCLC is a high-grade NEN characterized by a biological aggressiveness like that of LCNEC, early spread to distant metastases or metastatic disease at the time of diagnosis, and a negative prognosis with a poor OS. As regards localized disease, median survival has been reported to be 15–20 months, and only 20%–25% of patients survived after 5 years from diagnosis. According with ESMO clinical practice guideline, chemotherapy with platinum and etoposide is the first-line approach, while as second-line treatment topotecan has been approved [66]. Among NENs, SCLC is one of the most studied tumours and data on its biology have led to possible new therapeutic options. Since now, a high TMB has been identified, opening the scenario of immunotherapy due to its predictive role as possible biomarkers of response. Moreover, even if SCLCs scarcely express immune-checkpoint molecules, ICIs with a promising clinical activity are currently under investigation [67]. In this view, immunotherapy has been evaluated in many trials exploring its role in different settings, such as first-line therapy or second- or further line, as well as in monotherapy or in combined schedules, with the scope of leading to an innovative management of this high-grade neoplasms.

#### Atezolizumab

The FDA approved few years ago atezolizumab in combination with carboplatin and etoposide as first-line treatment of adult patients with extensive-stage (ES) SCLC. Approval was based on the IMpower133 randomized, multicentre, double-blind, placebo-controlled trial enrolling more than 400 patients with ES SCLC who received no prior chemotherapy for extensive stage disease. Atezolizumab plus carboplatin and etoposide, for a maximum of 4 cycles, followed by atezolizumab as maintenance until disease progression or unacceptable toxicity, were compared to placebo and carboplatin and etoposide for a maximum of 4 cycles, followed by placebo until disease progression or unacceptable toxicity. Efficacy was measured by an outcome showing a median OS of 12.3 months (10.8, 15.9) for patients receiving atezolizumab with chemotherapy and 10.3 months (9.3, 11.3) for those receiving placebo with chemotherapy (hazard ratio 0.70; 95% CI: 0.54, 0.91; p = 0.0069). Median PFS was 5.2 months (4.4, 5.6) compared with 4.3 months (4.2, 4.5) in the atezolizumab and placebo arms, respectively (HR 0.77; 0.62, 0.96; p = 0.0170). The most common adverse reactions, reported in ≥ 20% of patients who received atezolizumab, were asthenia, nausea, alopecia, constipation and decreased appetite [68].

Furthermore, atezolizumab versus conventional chemotherapy has been investigated as second-line therapy in 73 patients prior treated with platinum-based chemotherapy. Patients were not selected considering PD-L1 expression. The primary endpoint was objective response rate at 6 weeks, experienced by 1 of 43 eligible atezolizumab patients (2.3%, 95% confidence interval [CI]: 0.0–6.8). Median PFS was 1.4 months (95% CI: 1.2–1.5) with atezolizumab and 4.3 months (95% CI: 1.5–5.9) with chemotherapy. OS did not significantly differ between groups. Median OS was 9.5 months versus 8.7 months for the atezolizumab and the chemotherapy group, respectively (adjusted hazard ratio atezolizumab: 0.84, 95% CI: 0.45–1.58; p = 0.60) [69].

#### Durvalumab

Durvalumab, an IgG1 kappa anti-PD-L1 monoclonal human antibody, in combination with etoposide and either carboplatin or cisplatin has been recently approved by FDA as first-line treatment of patients with ES-SCLC. Approval was based on data from CASPIAN randomized, multicentre, active-controlled, open-label trial. Patients with untreated ES-SCLC were randomized to durvalumab plus chemotherapy vs. chemotherapy alone. The compelling result was concerning OS with a median of 13.0 months (95% CI: 11.5, 14.8) in the durvalumab plus chemotherapy arm compared with 10.3 months (95% CI: 9.3, 11.2) in the chemotherapy alone arm (hazard ratio 0.73; 95% CI: 0.59, 0.91; p = 0.0047). PFS (96% of total planned events) showed a HR of 0.78 (95% CI: 0.65, 0.94), with median PFS of 5.1 months (95% CI: 4.7, 6.2) in the durvalumab plus chemotherapy arm and 5.4 months (95% CI: 4.8, 6.2) in the chemotherapy alone arm. ORR was 68% (95% CI: 62%, 73%) in the durvalumab plus chemotherapy arm and 58% (95% CI: 52%, 63%) in the chemotherapy alone arm. Adverse reactions experienced by more than 20% of patients with ES-SCLC were nausea, asthenia and alopecia [70].

Moreover, the CASPIAN phase III trial also evaluated the combined therapy with durvalumab plus tremelimumab, an anti-CTLA-4 human monoclonal IgG2 antibody, plus platinum-etoposide followed by durvalumab as maintenance, until disease progression or unacceptable toxicity. Results from this combined therapy are not available yet [71].

#### Ipilimumab

The efficacy and safety of ipilimumab, an anti-CTLA4 inhibitor, was investigated in a randomized, double blind phase III trial without any evidence of prolonged OS with the combination of ipilimumab with chemotherapy. Indeed, 1132 patients with a diagnosis of ES-SCLC received as first-line ipilimumab or placebo plus etoposide and platinum. Median OS was 11.0 months for chemotherapy plus ipilimumab versus 10.9 months for chemotherapy plus placebo (hazard ratio, 0.94; 95% CI, 0.81 to 1.09; P = 0.3775). Median PFS was 4.6 months in the chemotherapy plus ipilimumab arm versus 4.4 months in the chemotherapy plus placebo arm (hazard ratio, 0.85; 95% CI, 0.75 to 0.97). Observed adverse effects were comparable in the 2 arms but it is worth highlighting that diarrhea, rash, and colitis, were more represented in the ipilimumab arm [72].

#### Nivolumab monotherapy or combined therapy Nivolumab plus Ipilimumab

As for first line-maintenance, the phase III trial CheckMate-451, enrolling 834 patients with not progressed ES-SCLC after first-line platinum-based chemotherapy, investigated nivolumab, an IgG4 anti-PD1 human monoclonal antibody, and the combined therapy with nivolumab plus ipilimumab. Primary endpoint was OS for nivolumab plus ipilimumab versus placebo and secondary endpoints included OS for nivolumab versus placebo. Initial results from a 2019 ESMO abstract did not show an improved survival with median OS of 10.4 vs. 9.2 vs. 9.6 months in the nivolumab, nivolumab combined with ipilimumab, and placebo arms, respectively. Considering the side effects observed, the higher rate of all grade of them was reported in the nivolumab plus ipilimumab arm (86%) [73].

As regards II-line approach, the open-label, randomized phase III trial CheckMate-331 failed to show an improved OS comparing nivolumab versus chemotherapy (topotecan or amrubicin) in patients with relapsing SCLC after first-line platinum-based chemotherapy. Indeed, median OS was 7.5 months in the nivolumab group and of 8.4 months in the control one. The median PFS was 1.5 vs. 3.8 months (HR 1.41 (95% CI: 1.18–1.69)) in the nivolumab and the topotecan arms, respectively. The evidence to pinpoint is the rate of adverse effect: all grade 55% in the nivolumab arm and 90% in the control arm [74]. Nivolumab alone and nivolumab plus ipilimumab in recurrent SCLC were investigated in the CheckMate-032 multicentre, open-label, phase 1/2 trial. Enrolled patients had at least already completed first-line platinum-based chemotherapy, and most of them also other line of therapy. The schedule as second or further approach was nivolumab or nivolumab plus ipilimumab for four cycles, followed by nivolumab until disease progression or unacceptable toxicity. ORR was 10% in patients treated with nivolumab monotherapy, 23% in the arm receiving nivolumab 1 mg/kg plus ipilimumab 3 mg/kg, and 19% in that receiving nivolumab 3 mg/kg plus ipilimumab 1 mg/kg [75].

Considering monotherapy as a third-line treatment, FDA approved nivolumab for patients with metastatic SCLC with progression after platinum-based chemotherapy and at least one other line of therapy. Approval was based on data concerning the ORR in a subgroup of patients from CheckMate-032. Indeed, 109 patients with metastatic SCLC, regardless of tumour PD-L1 expression, were treated with nivolumab monotherapy in a third-line setting. The ORR was 12% (95% CI: 6.5, 19.5). Responses were durable for 6 months or longer in 77%, 12 months or longer in 62%, and 18 months or longer in 39% of the 13 responding patients. PD-L1 tumour status seemed not to predict response in this cohort. As for safety, serious adverse reactions occurred in 45% of patients. The most frequent (≥ 2%) serious events were pneumonia, dyspnoea, pneumonitis, pleural effusion, and dehydration [76].

#### Pembrolizumab

Pembrolizumab is an IgG4 anti-PD-1 monoclonal antibody. Its efficacy and safety in patients with SCLS were evaluated in a second-line approach. An interim analysis of the KEYNOTE-158 (a phase 2 basket study comprizing 11 different cancer types) investigated pembrolizumab in 107 patients with advanced SCLC treated with chemotherapy as first-line and evidence of disease progression or intolerance to the aforementioned standard therapy. Tumor expression of PD-L1 was also assessed. Median OS was 9.1 months (95% CI, 5.7–14.6), 14.6 months in patients with PD-L1–positive tumors, and 7.7 months (95% CI, 3.9–10.4) neoplasms without PD-L1 expression. As results, pembrolizumab has to be further investigated, especially in patients with advanced SCLC and tumor samples positive for PD-L1 expression [77].

FDA approved pembrolizumab for patients with metastatic SCLC and evidence of disease progression on or after platinum-based chemotherapy and at least one other prior line of therapy. Approval was based on data derived from a pooled analysis gathering 83 patients from the SCLC group of both KEYNOTE-158 and KEYNOTE-028 trials. Patients received pembrolizumab until documented disease progression, unacceptable toxicity, or a maximum of 24 months. ORR was 19% (95% CI: 11, 29). The CR rate was 2% and responses were durable for 6 months or longer in 94%, 12 months or longer in 63%, and 18 months or longer in 56% of the 16 responding patients. The drug was discontinued for adverse reactions in 9% of patients and serious adverse events (AEs) occurred in 31% [78].

Furthermore, pembrolizumab was investigated combined with paclitaxel in 26 patients with etoposide/platinum-refractory ES-SCLC showing a moderate activity with acceptable toxicity. ORR was 23.1%, median PFS and OS were 5.0 months (95% CI: 2.7–6.7) and 9.1 months (95% CI: 6.5–15.0), respectively [41].

## Merkel cell carcinoma

MCC is a rare skin malignant tumor first described in 1972 [79], which neuroendocrine origin has been hypothesized only in 2012 [80]. Although being a rare neoplasm, it is responsible for the higher number of skin cancer-related death per year after melanoma. About 80% of the cases is driven by the infection of Merkel cell polyomavirus (MCPyV) [81], therefore being the major risk factor, while other risk factors for the development of MCC include UV irradiation, and immunodeficiency conditions.

Since patients showing a particularly strong immune response have been found to have more favorable prognoses [82], the possibilities of immunotherapy for MCC appear to be greater than in other types of neoplasms, including other NENs.

Indeed, therapy with ICIs for MCC is certainly proceeding at a faster pace than for the majority of other NENs, and evidence from literature concerning MCC is more robust.

The position of immunotherapy in the treatment algorithm of metastatic/advanced MCC is beginning to gain more and more consideration, since chemotherapy, despite showing satisfying response rates, cannot guarantee a durable response [83]. Considering that some chemotherapy regimens can further worsen the impairment of the patient’s immune system, and given the pivotal role of such factor when speaking of MCC, attention towards different approaches is steadily growing, and ICIs are drawing much of such attention.

As said, the risk of developing MCC is increased from five to 50-fold in persons with proven T-cell dysfunction, such as HIV-positive patients, solid organ transplant recipients, patients affected by hematological malignancies, and other immune system-impairing situations. Indeed, immunocompromised individuals represent approximately 10% of all the MCC patients [82, 84, 85]. Furthermore, immunosuppression represents a dramatic negative prognostic factor for mean survival, with patients showing any degree of immune system dysfunction showing an OS at three years approximately half of immunocompetent individuals (40% vs 74%) [86]. Conversely, better outcomes have been observed for patients showing particularly strong immune response features, such as highly CD8^+^ lymphocytes infiltrated tumors and MCCs showing no primary (MCC of unknown primary), which have higher tumor mutational burden and drive strong immune response [87, 88].

It comes of no surprise, though, due to its peculiar carcinogenesis, that MCC is indeed a highly immunogenic neoplasm.

As a matter of fact, it has been observed that both the infection by MCPyV and the DNA damage from UV radiation exposure (and the consequent high tumor mutational burden) lead to an increased response by the patient’s immune system [89].

MCPyV-positive MCCs express virus-related oncoproteins that eventually will drive unregulated growth [90]. Such proteins cause the patient’s immune system response in terms of intratumoral infiltration of CD8 + lymphocytes, the presence of MCPyV-specific T cells in the peripheral blood stream and IgG antibodies against said antigens [91, 92].

Similar pattern has been shown also in virus-negative MCCs, where the significant mutational burden caused by UV-radiation leads to production of neo-epitopes and proteins that are recognized by the patient immune system [93]. The presence of the aforementioned markers of immune system activation are all predictors of better prognosis [82].

Where it is true that a robust immune system activation is a favorable prognostic factor, it is also true that MCC can develop methods to escape the patient’s immune system. For example, MCCs thwart NK and T-cell–mediated oncolysis is by downregulating Toll-like receptor 9 MICA/MICB and MHC. A pivotal role in escaping lymphocyte-mediated eradication is also represented by PD-1 signaling: the tumor microenvironment, through the action of type II interferons, cause the overexpression of PD-L1 by the cancer cells, contributing to a decrease in the ability of inducing tumor cell death. Moreover, CD8 + T-cells infiltrating the tumor frequently overexpress PD-1 protein in response to persistent viral or tumor antigen exposure, as a marker of T-cell exhaustion [94].

PD-1 signaling appears to play a fundamental role in MCC; moreover, the highly immunogenic nature of this tumor provides a credible rationale for treatment with ICIs. To date, the only two ICI molecules FDA-approved for MCC are avelumab and pembrolizumab. [95, 96]; nevertheless, studies and trials concerning different ICIs are starting to populate the scientific panorama and initial evidence is available also for them eligible papers are summarized in Table 2.

**Table 2:** Published and ongoing RCT for ICIs in MCC

First name(s)	Molecule(s)	Full Title	Year	Journal	Patients, n	Type of study	Primary outcome(s)
Kaufman HL*	Avelumab	Avelumab in patients with chemotherapy-refractory metastatic Merkel cell carcinoma: a multicentre, single-group, open-label, phase 2 trial	2016	Lancet Oncol	88	multicentre, prospective, open-label clinical trial	Best Overall Response Durable Response Rate
Topalian SL	Nivolumab	[ABSTRACT] Non-comparative, open-label, multiple cohort, phase 1/2 study to evaluate nivolumab (NIVO) in patients with virus associated tumors (CheckMate 358): Efficacy and safety in Merkel cell carcinoma (MCC)	2017	Cancer Res	25	multicentre, prospective, open-label clinical trial	Objective Response Rate Safety
Kaufman HL*	Avelumab	Updated efficacy of avelumab in patients with previously treated metastatic Merkel cell carcinoma after 1 year of follow-up: JAVELIN Merkel 200, a phase 2 clinical trial	2018	J Immunother Cancer	88	*	*
D’Angelo SP**	Avelumab	Efficacy and safety of first-line avelumab treatment in patients with stage IV metastatic merkel cell carcinoma	2018	JAMA Oncol	39	multicenter, single-arm, open-label clinical trial	Objective Response Rate Duration of Response Safety
Nghiem P	Pembrolizumab	Durable tumor regression and overall survival in patients with advanced Merkel cell carcinoma receiving pembrolizumab as first-line therapy	2019	J Clin Oncol	50	multicenter, non-randomized, open-label clinical trial	Objective Response Rate Progression-free Survival Overall Survival Adverse Events
Knepper TC	Avelumab or Pembrolizumab or Nivolumab or Ipilimumab + Nivolumab	The Genomic Landscape of Merkel Cell Carcinoma and Clinicogenomic Biomarkers of Response to Immune Checkpoint Inhibitor Therapy	2019	Clin Cancer Res	36	retrospective	Response Rate
LoPiccolo J	various	Rescue therapy for patients with anti-PD-1-refractory Merkel cell carcinoma: a multicenter, retrospective case series	2019	J Immunother Cancer	13	retrospective case series	Objective Response
Topalian SL	Nivolumab	Neoadjuvant Nivolumab for Patients With Resectable Merkel Cell Carcinoma in the CheckMate 358 Trial	2020	J Clin Oncol	39	non-comparative, open-label clinical trial	Objective Response Rate Incidence of Adverse Events Rate of surgery delay
Spassova I	Avelumab or Pembrolizumab or Nivolumab	Predominance of central memory T cells with high Tcell receptor repertoire diversity is associated with response to PD-1/PD-L1 inhibition in Merkel cell carcinoma	2020	Clin Cancer Res	41	retrospective	Overall Response
Bystrup Boyles T	Pembrolizumab	Pembrolizumab as first line treatment of Merkel cell carcinoma patients—a case series of patients with various co-morbidities	2020	Acta Oncol	7	prospective	Objective Response Rate Incidence of Adverse Events Overall Survival Progression-free Survival
Levy S	Avelumab	Avelumab for advanced Merkel cell carcinoma in the Netherlands: a real-world cohort	2020	J Immunother Cancer	54	multicenter, retrospective	Response Rate Duration of Response
Walker JW	Avelumab	Efficacy and safety of avelumab treatment in patients with metastatic Merkel cell carcinoma: experience from a global expanded access program	2020	J Immunother Cancer	240	retospective	Response Rate
Glutsch V	Ipilimumab + Nivolumab	Activity of ipilimumab plus nivolumab in avelumab-refractory Merkel cell carcinoma	2021	Cancer Immunol Immunother	5	retospective	Response Rate Incidence of Adverse Events

*Studies comprising the same patients, part of Javeli trial part a

**Javelin part b

### Avelumab

This drug is rather unique amongst the ICIs since, beside its anti-PD-1/PD-L1 activity, it can mediate antibody-dependent cell-mediated cytotoxicity (ADCC). Anyway, no proved lysis of activated immune cells from avelumab has been observed so far [95, 97]. It was granted accelerated approval by FDA specifically for the treatment of metastatic MCC in adults and pediatric patients at least 12 years old, regardless of previous chemotherapy, subsequently to results produced in the JAVELIN Merkel 200 trial. In this study 88 patients with stage IV chemotherapy-refractory MCC were enrolled and treated with avelumab. ORR was 33.0% and responses were found to be durable, with 74% lasting more than 1 year. ORR was associated with a 94% risk reduction of death at 18 months. Beside such consistent results, avelumab was well tolerated, with only 5% of the treated patients developing grade 3 AEs and no patients experiencing a grade 4 AEs, with an improved quality of life. [98, 99] As expected, patients with PD-L1 expressing neoplasms had better response [100].

Following these findings, initial results from an interim analysis of the ongoing phase III JAVELIN Merkel 200 trial part B where avelumab was used for MCC as first line therapy were released in 2018, showing an ORR of 62.1% with 14 of 18 responses (77.8%) ongoing at the time of analysis. In the responding group, the estimated duration of response of at least 6 months was a remarkable 83%, with no grade 4 or 5 adverse event registered [101].

Real life experience is so far limited, nevertheless evidences have already been produced by Walker et al. [102] in their study including almost 500 patients with by either MCC or progressive MCC. The authors confirmed the positive results seen in the JAVELIN trial. In fact, among 240 evaluable patients, ORR was 46.7% and DCR 71.2%, with almost 23% of CR. Mean treatment duration was 7.9 months and, surprisingly, out of 16 immunocompromized patients, 11 (68.8%) experienced DCR, and ORR was (37.5%). These results were comparable to those recorded in the non-immunocompromized cohort, therefore opening a potential new window also for this category.

### Pembrolizumab

Recently, in late 2018, pembrolizumab gained approval by FDA for treatment of recurrent locally advanced or metastatic MCC, based on the KEYNOTE-017 trial, a multicenter, nonrandomized, open-label trial that enrolled 50 patients with recurrent locally advanced or metastatic MCC who had not received prior systemic therapy for their advanced disease [103, 104].

In KEYNOTE-017 trial 50 patients with recurrent locally advanced or metastatic MCC and no prior systemic chemotherapy were enrolled. According to the protocol, patients received pembrolizumab as a first-line treatment for 2 years or until PD or withdrawal for toxicity. Of such cohort, 68% had PD-L1 positive neoplasms. Overall response rate was 56%, with 24% experiencing CR and 32% PR, with ORRs of 59% in virus-positive and 53% in virus-negative tumors at a median follow-up time of 14.9 months.

While in the avelumab trial patients with tumor expressing PD-L1 displayed a better response, in the pembrolizumab study such a difference was not significant, however a positive trend in this direction was observed.

### Nivolumab

The results of nivolumab from clinical trials in patients with MCC are only partial so far and are mostly derived from the CheckMate-358 study (NCT02488759, Non-Comparative, Open-Label, Multiple Cohort, Phase 1/2 Study of Nivolumab Monotherapy and Nivolumab Combination Therapy in Subjects With Virus-Positive and Virus-Negative Solid Tumors).

In 2017, an abstract containing data from 25 patients enrolled was released [105]. The response, evaluable in 22 out of 25 patients (71% treatment-naïve patients), showed an ORR of 68%. At three months, PFS and OS rates were 82% and 92%, respectively, with 3/4 AEs occurring in 20% of patients (and in 12% of the patients led to therapy discontinuation).

More recently, in 2020, a paper, reporting the results of a neoadjuvant treatment in a cohort of MCC- patients (enrolled in the CheckMate 358), was published [106]. Out of the 36 patients who did undergo surgery, 47% achieved CR and 55% of the 33 radiographically evaluable patients who underwent surgery had at least tumor reduction ≥ 30%. Recurrence-free survival (RFS) and OS were not reached at 20.3 months.

The CheckMate-358 study is still ongoing, with its estimated completion date set for August 2022.

### Real-life Data

Such dazzling results from clinical trials, the most interesting one being probably the sustained durability response (especially when compared to chemotherapy), together with consistent response rates and acceptable safety profiles, already gained ICIs approval as recommended first-line treatments for metastatic MCC in countries such as US and Germany [107].

While initial evidence is available from clinical trials, fewer real-life data are available on ICIs in MCC patients. Few authors have described experience with such drugs in MCC so far [108–111]. In the mentioned real-life studies, which comprise cohorts of patients treated with either avelumab, pembrolizumab or nivolumab plus ipilimumab, response rates ranged between 39–57%, with the best one found in a cohort of only avelumab-treated patients [111]. Some authors also included immunocompromised patients in their studies. Knepper et al. [108] found significant relationship between the number of lines of therapy and response, with a 75% rate for first-line, 39% and 18% in second and third, respectively. Spassova et al. [109] in their retrospective study found that immunocompetent patients responded better than ones with hematological malignancies or under immunosuppressive drugs, whereas the type of drug was not found to be predictor of response. Levy et al. [111] showed that for avelumab, real-life data appear to be in line with results from clinical trials in terms of efficacy and toxicity. DOR in their cohort was 8.4 months, but with 43% of the patients having ongoing response at the end of the study. In the small retrospective series of patients treated with first-line pembrolizumab reported by Bystrup Boyles et al. [110], DOR was confirmed to be consistent, ranging from 16.3 to 27.1 months at cut off, but toxicity was found to be much higher compared to clinical trials data.

Data of efficacy of first-line ICI-based therapy appear to be promising, but still a consistent portion of patients either do not respond to the chosen ICI or develop resistance. Since chemotherapy does not provide a sustained response over time, the possibility, after failure of the first one, of a second-line ICI-therapy with a different target is definitively a possibility worth to be discussed. So far, few studies have investigated said option: LoPiccolo et al. [112] reported a case series of 13 patients treated with ICIs after experiencing PD under anti-PD-1 therapy. OR was observed in 31% of patients, with one patient refractory to anti-PD-1 therapy experiencing tumor regression with a PD-L1 antibody. Glutsch et al. [113] in their paper report that 3 out of 5 avelumab-refractory patients responded to the combination of ipilimumab plus nivolumab after failure of avelumab-based therapy, experiencing no grade 3 or 4 AEs.

## Conclusions

Immunotherapy, at this time, finds its precise place in the treatment of SCLC and MCC. In these two forms of aggressive and high-grade NENs, ICIs have obtained important and encouraging results, which in the future will still be implemented by the ongoing trials regarding combined treatments among different ICIs and other molecules.

On the contrary, with regard to the well-differentiated forms of NENs, the results obtained so far have been disappointing and, unfortunately, immunotherapy cannot be considered a promising therapy at the moment. ICIs have no impact on clinical practice in the treatment of differentiated NENs and do not yet have a precise role in the therapeutic sequence of these tumors.

Considering the low TMB and the scarce tumor-infiltrating lymphocytes that characterize NETs, these results, obtained so far, are not surprising. This picture does not mean that in the future a better outcome cannot be obtained in patients with NETs as well. However, the most important aspect will be to study strategies that can make NETs more susceptible to response to ICI and, thus, enhance the effectiveness of these treatments. Therefore, the combination of conventional therapy, target therapy and immunotherapy deserves attention and warrant to be explored [114]

A sequential chemotherapy, possibly inducing an increase in TMB and tested before immunotherapy, could be a hypothesis deserving more consideration. Radiotherapy or PRRT, that precedes the start of ICI and induces an inflammation at tumoral level, with an increase of tumor-infiltrating lymphocytes, could be another approach to explore. Equally essential will be the identification of biomarkers useful for selecting patients potentially responsive to this type of treatment. In fact, responses to immunotherapy were also reported in tumors presented with low PD-L1 expression and low TMB, underlying that a screening of patients based only on these two biomarkers could exclude potential responders to ICIs [32, 33].

Some intrinsic characteristics of NENs make difficult to study all these hypotheses, since the large heterogeneity of NENs in their biology and clinical behavior, and their relative rarity, combined with the habit of conducting studies on non-homogeneous case-series by primitive site or grading, do not allow to obtain univocal and established data on this topic. Until we have clarified all these aspects, it will be difficult for ICI to revolutionize the therapeutic approach of well-differentiated NENs.

## Abbreviations

CTLA-4: Cytotoxic T-Lymphocyte Antigen 4
PD-1: Programmed death-1
PD-L1: Programmed death-1 ligand
ICIs: Immune checkpoint inhibitors
WHO: World Health Organization
FDA: Food and Drug Administration
ESMO: European Society for Medical Oncology
IHC: Immunohistochemical
NENs: Neuroendocrine neoplasms
NECs: Neuroendocrine carcinomas
NETs: Neuroendocrine tumors
GEP: Gastroeneteropancreatic
MCC: Merkel cell carcinoma
SCLC: Small cell lung cancer
LCNEC: Large cell neuroendocrine carcinoma
GI-NENs: Gastrointestinal neuroendocrine neoplasms
panNENs: Pancreatic neuroendocrine neoplasms
TC: Typical Carcinoid
AC: Atypical Carcinoid
MCPyV: Merkel cell polyomavirus
SRLs: Somatostatin receptor ligands
PRRT: Peptide receptor radionuclide therapy
TKI: Tirosin Kinase Inhibitors
TMB: Tumor mutational burden
Treg: Regulatory T cells
ADCC: Antibody-dependent cell-mediated cytotoxicity
RCTs: Randomised Controlled Trials
CBR: Clinical benefit rate
DCR: Disease control rate
CR: Complete response
PR: Partial response
PD: Progressive disease
PFS: Progression-free survival
RFS: Recurrence-free survival
OS: Overall survival
ORR: Objective response rate
DOR: Durability of response
ES: Extensive-stage
AEs: Adverse events
